# Supplementary material for: Cohort profile: Mothers who use substances and their children in British Columbia, Canada
Source: PLoS One. 2026 May 26;21(5):e0348262. doi: 10.1371/journal.pone.0348262 (PMC13210378; doi:10.1371/journal.pone.0348262)
Supplement: S1 Fig — MSP, Medical Services Plan; DAD, Discharge Abstract Database; BCVS, BC Vital Statistics; PNET, Pharmanet; PSBC, Perinatal Services BC; SDPR, BC Social Development and Poverty Reduction; NACRS, National Ambulatory Care Reporting System; BCCS, BC Coroners Service; BC Corrections, BC Provincial Corrections. (DOCX) [file pone.0348262.s001.docx]

# **S1 Figure. Data collection timeline for each provincial administrative database (01/01/1996-31/12/2022)**

**
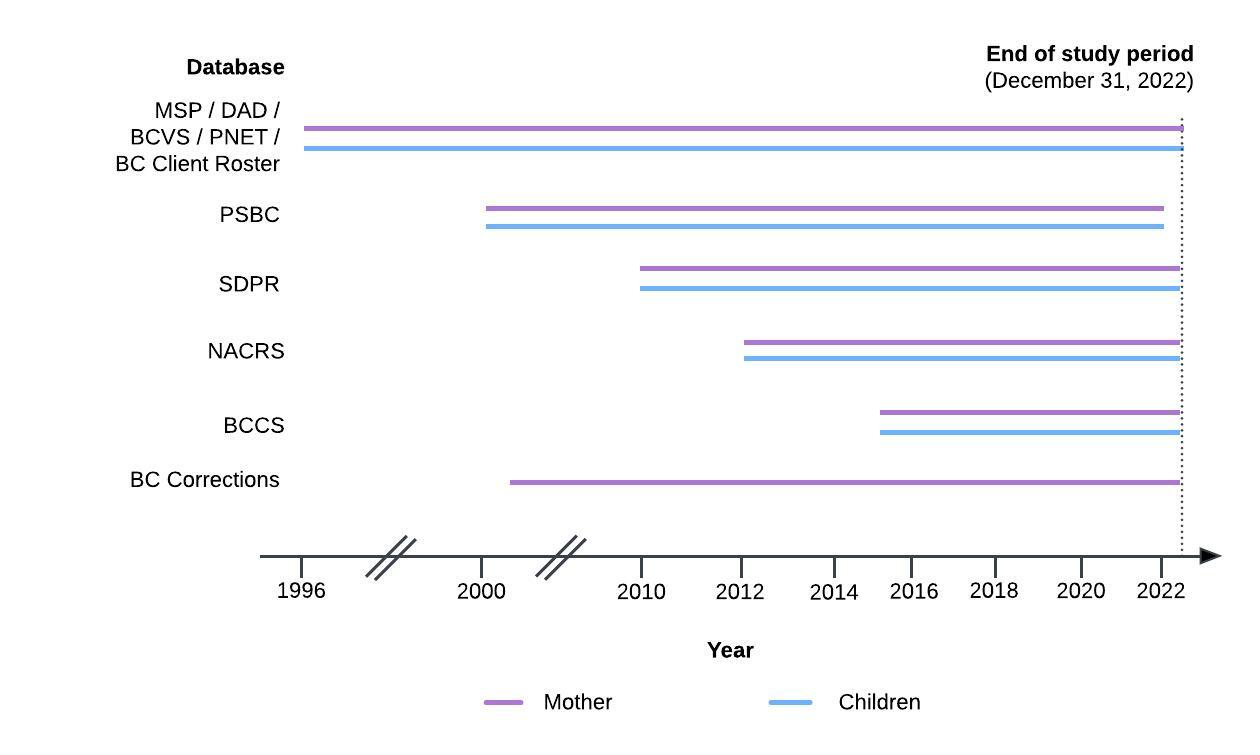
**

MSP, Medical Services Plan; DAD, Discharge Abstract Database; BCVS, BC Vital Statistics; PNET, Pharmanet; PSBC, Perinatal Services BC; SDPR, BC Social Development and Poverty Reduction; NACRS, National Ambulatory Care Reporting System; BCCS, BC Coroners Service; BC Corrections, BC Provincial

Corrections
