# Supplementary material for: Cohort profile: Mothers who use substances and their children in British Columbia, Canada
Source: PLoS One. 2026 May 26;21(5):e0348262. doi: 10.1371/journal.pone.0348262 (PMC13210378; doi:10.1371/journal.pone.0348262)
Supplement: S1 Table — Abbreviations: DIN: Drug Identification Number; PIN: Product Identification Number; † Coding structures used by the Canadian Institute of Health Information. (DOCX) [file pone.0348262.s002.docx]

**S1 Table. Description of ten provincial administrative database in British Columbia, 2000-2022**

| **Database** | **Description** | **Generating process** | **Key content** |
| --- | --- | --- | --- |
| PharmaNet[1] | All prescriptions for drugs and medical supplies dispensed from pharmacies including hospital outpatient dispensations. | Electronically submitted by pharmacists dispensing medications in real time. Required for reimbursement. | Drugs dispensed (using DIN/PIN number), date of dispensation, quantity and duration of prescription, billing information, **prescriber code** and drug costs. |
| Discharge Abstract Database (DAD)[2] | All hospital discharges, day surgery, transfers, and deaths of inpatients. Data of BC residents treated at hospital out of province, and out-of-province residents treated within BC hospitals included. | Data files grouped into fiscal years by separation date (not admission date). Each hospital submits electronic records of client visits to the provincial government which cleans and then submits the records to the Canadian Institute for Health Information (CIHI). CIHI regularly conducts re-abstraction to ensure data quality. | Hospitalization dates, most responsible diagnosis (ICD-9/-10-CA code) and up to 24 additional diagnostic codes, 25 procedure codes using CCI/CCP procedure/ intervention codes^†^, transport method, transfers, **primary physician responsible** for stay, condition specific resource intensity weights, inpatient grouping. Hospital number, level of care, admission date/time, admission category, readmission, and transfer codes, discharge date/time, discharge, disposition, length of stay, stay by level of care. |
| Medical Services Plan (MSP) Database[3] | All medically necessary services provided by fee-for-service practitioners covered by the province’s universal insurance program: Medical Services Plan (MSP). | Majority of billing records submitted electronically by practitioners’ offices for reimbursement purposes. Diagnosis codes accurate only to 3^rd^ digit. | Medically necessary services including laboratory and diagnostic procedures (x-rays, ultrasounds), and dental and oral surgery performed in hospital. Up to 5 diagnoses codes included (ICD-9-CA). Service date, fee item, diagnostic codes, **practitioner code**, service costs and location. |
| Vital Statistics (VS)[4] | All deaths registered in the province. | Data is checked against nationally uniform vital registration and statistics standards. | Date of death (year and month), location, underlying cause of death (ICD-9/-10-CA), and nature of injury codes. |
| National Ambulatory Care Reporting System (NACRS)[5] | All emergency department visits | The centralized data processing of the NACRS records, done by the Canadian Institute of Health Information (CIHI), results in increased efficiency and  standardization among the participating provinces. | Institution/hospital number; triage, registration, assessment and disposition date/time; presenting complaint codes; ED discharge diagnosis code. |
| Perinatal Services BC (PSBC)[6] | Maternal and child health for all provincial births | Perinatal data is collected from facilities throughout the province and imported into the central BC Perinatal Data  Registry (BCPDR). | Demographics of mother, past obstetric history, information on current pregnancy, labour and delivery, post-delivery, diagnoses and procedures; demographics of baby, information on birth and care, diagnoses and procedures |
| BC Social Development and Poverty Reduction  (SDPR)[7] | Income Assistance benefits for qualifying people in British Columbia | Data is extracted from the Ministry of Social Development and Poverty Reduction’s records of social assistance or disability assistance payments that have been made to individuals under the BC Employment and Assistance program. | Program name, age, family and dependent information, total payment received, indicators for no fixed address, methadone use, crisis grants, and other income received. |
| BC Provincial Corrections[8] | Individuals incarceration in provincial prisons (sentences of ≤ 2 years) | All adults corrections data received from the Ministry of Public Safety and Solicitor General (PSSG) | Inmate client information, dates and times of admissions and release |
| BC Coroners Service  (BCCS)[9] | All unnatural, sudden and unexpected, unexplained or unattended deaths in British Columbia | The agency maintains a database and conducts ongoing surveillance of common causes and circumstances of death. | Date of death, location of injury, cause of unnatural death, mode of consumption, post mortem toxicology |
| BC Client Roster[10] | Demographic and geographic information for the Ministry’s clients. | The primary source for the Client Roster is the Ministry’s Client Registry System: Enterprise Master Patient Index (EMPI). Other sources include Registration and Premium Information Determination (RAPID), formerly Registration and Premium Billing information (R&PB), which provides information on MSP eligibility and client event data. Processing resolves multiple individual identifiers to a single identity, and consolidates demographic information from a variety sources to single “best” values. | Client Study ID, client gender, birth year and month, health authority, health service delivery area, local health area, community health service area, forward sortation area. |

*Abbreviations:* DIN: Drug Identification Number; PIN: Product Identification Number; ^†^ Coding structures used by the Canadian Institute of Health Information

**S1 Table References**

1. British Columbia Ministry of Health [creator] (2024): PharmaNet. British Columbia Ministry of Health [publisher]. Data Extract. MOH (2024). <http://www2.gov.bc.ca/gov/content/health/conducting-health-research-evaluation/data-access-health-data-central>.

2. Canadian Institute of Health Information [creator] (2024): Discharge Abstract Database (Hospital Separations). British Columbia Ministry of Health [publisher]. Data Extract. MOH (2024). <http://www2.gov.bc.ca/gov/content/health/conducting-health-research-evaluation/data-access-health-data-central>.

3. British Columbia Ministry of Health [creator] (2024): Medical Services Plan (MSP) Payment Information File. British Columbia Ministry of Health [publisher]. Data Extract. MOH (2024). <http://www2.gov.bc.ca/gov/content/health/conducting-health-research-evaluation/data-access-health-data-central>.

4. British Columbia Ministry of Health [creator] (2024): Vital Statistics Deaths. British Columbia Ministry of Health [publisher]. Data Extract. MOH (2024). <http://www2.gov.bc.ca/gov/content/health/conducting-health-research-evaluation/data-access-health-data-central>. In: [creator] BCMoH, editor.

5. British Columbia Ministry of Health [creator] (2024): National Ambulatory Care Reporting System (NACRS). British Columbia Ministry of Health [publisher]. Data Extract. MOH (2024). <http://www2.gov.bc.ca/gov/content/health/conducting-health-research-evaluation/data-access-health-data-central>.

6. Perinatal Services BC [creator] (2024): British Columbia Perinatal Data Registry. British Columbia Ministry of Health [publisher]. Data Extract. MOH (2024). <http://www2.gov.bc.ca/gov/content/health/conducting-health-research-evaluation/data-access-health-data-central>.

7. British Columbia Ministry of Social Development and Poverty Reduction [creator] (2024): Social Development and Poverty Reduction Database (SDPR). British Columbia Ministry of Health [publisher]. Data Extract. MOH (2024). <http://www2.gov.bc.ca/gov/content/health/conducting-health-research-evaluation/data-access-health-data-central>.

8. Ministry of Public Safety and Solicitor General (PSSG) [creator] (2024): BC Corrections Dataset. British Columbia Ministry of Health [publisher]. Data Extract. MOH (2024). <http://www2.gov.bc.ca/gov/content/health/conducting-health-research-evaluation/data-access-health-data-central>.

9. BC Coroners Service [creator] (2024): BC Coroners Dataset. British Columbia Ministry of Health [publisher]. Data Extract. MOH (2024). <http://www2.gov.bc.ca/gov/content/health/conducting-health-research-evaluation/data-access-health-data-central>.

10. British Columbia Ministry of Health [creator] (2024). Client Roster. British Columbia Ministry of Health [publisher]. Data Extract. MOH (2024). <http://www2.gov.bc.ca/gov/content/health/conducting-health-research-evaluation/data-access-health-data-central>.
