# Supplementary material for: Cohort profile: Mothers who use substances and their children in British Columbia, Canada
Source: PLoS One. 2026 May 26;21(5):e0348262. doi: 10.1371/journal.pone.0348262 (PMC13210378; doi:10.1371/journal.pone.0348262)
Supplement: S2 Table — AHFS: American Hospital Formulary Service by the American Society of Health-System Pharmacists; BCPDR: British Columbia Perinatal Data Registry; DAD: Discharge Abstract Database (hospitalizations); DIN: drug identification number in PharmaNet (drug dispensations); ICD-9-CA: International Classification of Diseases, Ninth Revision, Canada. ICD-10-CA: International Statistical Classification of Diseases and Related Health Problems, Tenth Revisions, Canada; MSP: Medical Services Plan; NACRS: National Ambulatory Care Reporting System (emergency department visits); PIN: product identification number in PharmaNet (drug dispensations); SDPR: Social Development and Poverty Reduction; VS: Vital Statistics database in British Columbia (death records). a Diacetylmorphine or hydromorphone with some restrictions based on prescriber, dispensing pharmacy and/or date. *Pharmacare Plan C (Income Assistance) provides full coverage of eligible prescription costs for B.C. residents receiving benefits and income assistance through the Ministry of Social Development and Poverty Reduction, or in the care of, or in an agreement with Ministry of Children and Family Services for children and youth. (DOCX) [file pone.0348262.s003.docx]

# **S2 Table. Case finding algorithm of substance use, comorbidities, other conditions, and social determinants of health**

| **Health conditions** | **Diagnosis code** | **Case finding algorithm** | **References** |
| --- | --- | --- | --- |
| Opioid use | ICD-9-CA from DAD, MSP, VS: 304.0, 304.7, 305.5, 965.0, E850.0-E850.2; ICD-10-CA from DAD, NACRS, VS: F11, (X42, X44, X62, X64, Y12, or Y14) & (T40.0, T40.1, T40.2, T40.3, T40.4, or T40.6); Fee item from MSP: 39, 15039,13013,13014,36521; Indication of heroin/opioid use as risk factor in pregnancy from BCPDR | 1 opioid agonist treatment drug dispensation, or ≥ 3 physician claims, or 1 hospital admission, or 1 emergency department visit, or 1 perinatal, or 1 death record | [1] |
| Alcohol use | ICD-9-CA from DAD and MSP: 291, 303, 305.0, 357.5, 425.5, 535.3, 571.0-571.3, 655.4, V65.42; ICD-10-CA from DAD and NACRS: F10, Z50.2, Z71.4, Z72.1, G31.2, G62.1, G72.1, I42.6, K29.2, K70, K86.0, O35.4; DINPIN from PharmaNet: 2293269, 2158655, 2213826, 2444275, 2451883, 2534, 2542, 2041375, 2041391, 66124089, 66124085, 66124087; Indication of alcohol use disorder during the pregnancy from BCPDR | 1 drug dispensation, ≥ 3 physician claims, or 1 hospital admission, or 1 emergency department visit, or 1 perinatal, or 1 death record | [2], [3] |
| Non-opioid or non-alcohol use | *Cannabis*: *ICD-9-CA: 304.3, 305.2; ICD-10-CA: F12, T40.7; Indication of marijuana use at any time during the pregnancy from BCPDR* | ≥ 3 physician claims, or 1 hospital admission, or 1 emergency department visit, or 1 perinatal, or 1 death record | [4], [2] |
|  | *Stimulants* (including cocaine): *ICD-9-CA: 304.2, 304.4, 305.6, 305.7, 969.7, 970, E854.2, E854.3; ICD-10-CA: F14, F15, T40.5; Indication of cocaine use at any time during the pregnancy from BCPDR* |  |  |
|  | *Sedatives and hypnotics* (including Benzodiazepines): ICD-9-CA: 304.1, 305.4, 969.4, 967, E851, E852, E853.2; *ICD-10-CA: F13, T42.3-T42.7* |  |  |
|  | *Hallucinogens*: *ICD-9-CA: 304.5, 305.3, 969.6, E854.1; ICD-10-CA: F16, T40.8, T40.9* |  |  |
|  | *Unspecified*: *ICD-9-CA: 292, 304, 304.6, 304.8, 304.9, 305, 305.8, 305.9, 648.3, 655.5, 969; ICD-10-CA: F19, T43.6, Z50.3, Z71.5, Z72.2; Indication of unspecified drug use as a risk at any time during the pregnancy from BCPDR* |  |  |
| Mental health conditions | ICD-9-CA from DAD and MSP: 295-298, 300, 301, 308, 309, 311, 314, 317-319, 760.71; ICD-10-CA from DAD and NACRS: F20-F25, F28-F34, F38-F43, F48, F60 - F61, F69 - F73, F78 - F79, F90, Q86.0; MSP additional diagnostic code 50B; Indication of mental health during the pregnancy from BCPDR | ≥ 3 physician claims, or 1 hospital admission, or 1 emergency department visit, or 1 perinatal, or 1 death record | [4], [5],[6] |
| Serious mental disorders | *Bipolar disorder*: *ICD-9-CA: 296.4, 296.5, 296.6, 296.7, 296.8; ICD-10-CA: F31;* | *Bipolar disorder/major depressive disorder*: 1 hospital admission, or 1 emergency department visit, or >=2 physician claims within 12 months, or 1 death record | Bipolar: [7]  Major depressive disorder: [8], [7]  Schizophrenia: [8], [9] |
|  | *Major depressive disorders: ICD-9-CA: 296.2, 296.3; ICD-10-CA: F32.1, F32.2, F32.9, F33.1, F33.2, F33.9* |  |  |
|  | *Schizophrenia: ICD-9-CA: 295; ICD-10-CA: F20.0, F20.1, F20.2, F20.5, F20.8, F20.9;* | *Schizophrenia*: 1 hospital admission, or 1 emergency department visit, or >=2 physician claims at least 30 days apart in two years, or 1 death record |  |
| Hepatitis C virus | ICD-9-CA from DAD and MSP: 070.4, 070.5, 070.7; ICD-10-CA from DAD and NACRS: B17.1, B18.2, B19.2; AHFS category from PharmaNet: 8:18.40 | ≥ 3 physician claims, 1 drug dispensation, or 1 hospital admission, or 1 emergency department visit, or 1 death record | [10], [11], [12] |
| HIV | ICD-9-CA from DAD and MSP: 042‐044, 079.53, 795.8, V08; ICD-10-CA from DAD and NACRS: B20‐B24, B97.35, F02.4, O98.7, Z21; MSP fee item: 13015, 13105, 33645, 36370 | ≥ 3 physician claims, or 1 hospital admission, or 1 emergency department visit, or 1 death record | [13] |
| Non-cancer chronic pain | ICD-9-CA from DAD and MSP: 338.2, 338.4, 307.80, 307.89, 338.0, 719.41, 719.45-719.47, 719.49, 720.0, 720.2, 720.9, 721.0-721.4, 721.6, 721.8, 721.9, 722, 723.0, 723.1, 723.3-723.9, 724.0-724.6, 724.70, 724.79, 724.8, 724.9, 729.0-729.2, 729.4, 729.5, 350, 352-357, 344.0, 344.1, 997.0, 733.0, 733.7, 733.9, 781; ICD-10-CA from DAD and NACRS: F45.4, G89.0, G89.2, G89.4, M08.1, M25.50, M25.51, M25.55-M25.57, M43.2-M43.6, M45, M46.1, M46.3, M46.4, M46.9, M47, M48.0, M48.1, M48.8, M48.9, M50.8, M50.9, M51, M53.1-M53.3, M53.8, M53.9, M54, M60.8, M60.9, M63.3, M79.0-M79.2, M79.6, M79.7, M96.1, G50, G52-G64, G82, G97, M89, R29 | ≥ 3 physician claims, or 1 hospital admission, or 1 emergency department visit, or 1 death record | [14] |
| Receipt of income assistance* | PharmaCare specialty plan from PharmaNet: C (Income Assistance) or receipt of income assistance payment from SDPR |  | [15] |
| Unstable housing | ICD-9-CA from DAD, MSP: V60.0, V60.1; ICD-10-CA from DAD: Z59.0, Z59.1 or 3 consecutive months of no fixed address from SDPR |  | [16] |
| Social determinants of health | *Economic*: *ICD-9-CA from DAD and MSP: V60.0 - V60.1, V60.5, V60.8 - V60.9, V62.0 - V62.2, V69.1, V71.3, 994.3, E90.4; ICD-10-CA from DAD: E40 - E46, E51.1 - E51.2, E51.9, E52 - E56, E58 - E61, E63 - E64, T73.0 - T73.1, T73.8 - T73.9, X58, Z56 - Z57, Z59.0 - Z59.1, Z59.4 - Z59.9, Z72.4* | ≥ 3 physician claims, or 1 hospital admission, or 1 death record | [17] |
|  | *Education*: *ICD-9-CA from DAD and MSP: V61.2, V62, V62.3, 995.5; ICD-10-CA from DAD: Z62.8 - Z62.9, Z62.0, Z62.2, Z55, Z73.4* |  |  |
|  | *Environment*: *ICD-9-CA from DAD and MSP: V15.4, V15.8, V61.1, V61.4, V71.8, Z77.0 - Z77.1, V87.0 - V87.1, V87.3, 984.0 - 984.1, 995.8, 984.8 - 984.9, E86.1, E86.6, E96; ICD-10-CA from DAD: T74.0- T74.3, T74.9, T56.0, T76.0 - T76.3, T76.9, Z58.8, Z63.0, Z63.7, Z65.4 - Z65.5, Z91.4* |  |  |
|  | *Social*: *ICD-9-CA from DAD and MSP: V60.3 - V60.4, V61, V61.0, V61.3 - V61.4, V61.8 - V61.9, V62.4 - V62.5, V62.8 - V62.9, V69.2 - V69.5, V69.8 - V69.9; ICD-10-CA from DAD: F43.9, Z60, Z63.0 - Z63.1, Z63.3 - Z63.9, Z65.0 - Z65.3, Z65.8 - Z65.9, Z72.8, Z73.2 - Z73.3, Z73.8 - Z73.9* |  |  |
|  | *Health* *care*: *ICD-9-CA from DAD: V63.8 - V63.9; ICD-10-CA from DAD: Z74.8 - Z74.9, Z75.3 - Z75.4, Z75.8 - Z75.9* |  |  |

AHFS: American Hospital Formulary Service by the American Society of Health-System Pharmacists; BCPDR: British Columbia Perinatal Data Registry; DAD: Discharge Abstract Database (hospitalizations); DIN: drug identification number in PharmaNet (drug dispensations); ICD-9-CA: International Classification of Diseases, Ninth Revision, Canada. ICD-10-CA: International Statistical Classification of Diseases and Related Health Problems, Tenth Revisions, Canada; MSP: Medical Services Plan; NACRS: National Ambulatory Care Reporting System (emergency department visits); PIN: product identification number in PharmaNet (drug dispensations); SDPR: Social Development and Poverty Reduction; VS: Vital Statistics database in British Columbia (death records)**.** ^a^ Diacetylmorphine or hydromorphone with some restrictions based on prescriber, dispensing pharmacy and/or date. *Pharmacare Plan C (Income Assistance) provides full coverage of eligible prescription costs for B.C. residents receiving benefits and income assistance through the Ministry of Social Development and Poverty Reduction, or in the care of, or in an agreement with Ministry of Children and Family Services for children and youth

**S2 Table References**

1. Piske M, Zhou H, Min JE, Hongdilokkul N, Pearce LA, Homayra F, et al. The cascade of care for opioid use disorder: a retrospective study in British Columbia, Canada. Addiction. 2020;115(8):1482-93. Epub 2020/02/06. doi: 10.1111/add.14947. PubMed PMID: 31899565.

2. Degenhardt L, Randall D, Hall W, Law M, Butler T, Burns L. Mortality among clients of a state-wide opioid pharmacotherapy program over 20 years: risk factors and lives saved. Drug Alcohol Depend. 2009;105(1-2):9−15. Epub 2009 Jul 15. doi: 10.1016/j.drugalcdep.2009.05.021.

3. Centers for Disease Control and Prevention. Alcohol-Related ICD Codes 2020 [cited 2020 August 10]. Available from: <https://www.cdc.gov/alcohol/ardi/alcohol-related-icd-codes.html>.

4. Quan H, Sundararajan V, Halfon P, Fong A, Burnand B, Luthi JC, et al. Coding algorithms for defining comorbidities in ICD-9-CM and ICD-10 administrative data. Medical care. 2005;43(11):1130–9.

5. Clark DO, Von Korff M, Saunders K, Baluch WM, Simon GE. A chronic disease score with empirically derived weights. Med Care. 1995;33(8):783–95.

6. Health Quality Ontario. Hospital admissions for a mental illness or an addiction 2017. Available from: <http://indicatorlibrary.hqontario.ca/Indicator/Detailed/Mental-health-addiction-admissions/EN>.

7. Davis KA, Sudlow CL, Hotopf M. Can mental health diagnoses in administrative data be used for research? A systematic review of the accuracy of routinely collected diagnoses. BMC Psychiatry. 2016;16:263. doi: 10.1186/s12888-016-0963-x. PubMed PMID: 27455845; PubMed Central PMCID: PMCPMC4960739.

8. BC Centre for Disease Control. Chronic Disease Dashboard: Case Definitions 2020 [cited 2022 March 10]. Available from: <http://www.bccdc.ca/health-professionals/data-reports/chronic-disease-dashboard#Case--Definitions>.

9. Kurdyak P, Lin E, Green D, Vigod S. Validation of a Population-Based Algorithm to Detect Chronic Psychotic Illness. Can J Psychiatry. 2015;60(8):362-8. doi: 10.1177/070674371506000805. PubMed PMID: 26454558; PubMed Central PMCID: PMCPMC4542516.

10. Shah H, Bilodeau M, Burak KW, Cooper C, Klein M, Ramji A, et al. The management of chronic hepatitis C: 2018 guideline update from the Canadian Association for the Study of the Liver. CMAJ. 2018;190(22):E677-E87. doi: 10.1503/cmaj.170453. PubMed PMID: 29866893.

11. Perrone V, Sangiorgi D, Buda S, Degli Esposti L. Disease progression and health care resource consumption in patients affected by hepatitis C virus in real practice setting. Clinicoecon Outcomes Res. 2016;8:591-7. Epub 20161014. doi: 10.2147/CEOR.S108288. PubMed PMID: 27789966; PubMed Central PMCID: PMCPMC5072570.

12. Kim D, Li AA, Gadiparthi C, Khan MA, Cholankeril G, Glenn JS, et al. Changing Trends in Etiology-Based Annual Mortality From Chronic Liver Disease, From 2007 Through 2016. Gastroenterology. 2018;155(4):1154-63.e3. Epub 20180901. doi: 10.1053/j.gastro.2018.07.008. PubMed PMID: 30009816; PubMed Central PMCID: PMCPMC6467699.

13. Nosyk B, Colley G, Yip B, Chan K, Heath K, Lima VD, et al. Application and validation of case-finding algorithms for identifying individuals with human immunodeficiency virus from administrative data in British Columbia, Canada. PloS one. 2013;8(1):e54416. Epub 2013/02/06. doi: 10.1371/journal.pone.0054416. PubMed PMID: 23382898; PubMed Central PMCID: PMC3557280.

14. Tian TY, Zlateva I, Anderson DR. Using electronic health records data to identify patients with chronic pain in a primary care setting. J Am Med Inform Assoc. 2013;20(e2):e275-80. Epub 20130731. doi: 10.1136/amiajnl-2013-001856. PubMed PMID: 23904323; PubMed Central PMCID: PMCPMC3861913.

15. British Columbia Ministry of Social Development and Poverty Reduction [creator] (2024): Social Development and Poverty Reduction Database (SDPR). British Columbia Ministry of Health [publisher]. Data Extract. MOH (2024). <http://www2.gov.bc.ca/gov/content/health/conducting-health-research-evaluation/data-access-health-data-central>.

16. Peterson R, Gundlapalli AV, Metraux S, Carter ME, Palmer M, Redd A, et al. Identifying Homelessness among Veterans Using VA Administrative Data: Opportunities to Expand Detection Criteria. PLoS One. 2015;10(7):e0132664. Epub 2015/07/14. doi: 10.1371/journal.pone.0132664. PubMed PMID: 26172386; PubMed Central PMCID: PMCPMC4501742.

17. Agarwal AR, Prichett L, Jain A, Srikumaran U. Assessment of Use of ICD-9 and ICD-10 Codes for Social Determinants of Health in the US, 2011-2021. JAMA Netw Open. 6. United States2023. p. e2312538.
