# Supplementary material for: Cohort profile: Mothers who use substances and their children in British Columbia, Canada
Source: PLoS One. 2026 May 26;21(5):e0348262. doi: 10.1371/journal.pone.0348262 (PMC13210378; doi:10.1371/journal.pone.0348262)
Supplement: Attachment — Submitted filename: Reviewer Comments for Manuscript PONE_final.docx [file pone.0348262.s005.docx]

**R&R for manuscript "Cohort Profile: Mothers who use substances and their children in British Columbia, Canada"**

Journal Requirements:

When submitting your revision, we need you to address these additional requirements.

1. Please ensure that your manuscript meets PLOS ONE's style requirements, including those for file naming. The PLOS ONE style templates can be found at
   <https://journals.plos.org/plosone/s/file?id=wjVg/PLOSOne_formatting_sample_main_body.pdf> and
   <https://journals.plos.org/plosone/s/file?id=ba62/PLOSOne_formatting_sample_title_authors_affiliations.pdf>

**RESPONSE: Done, as requested.**

2. We note that you have indicated that there are restrictions to data sharing for this study. For studies involving human research participant data or other sensitive data, we encourage authors to share de-identified or anonymized data. However, when data cannot be publicly shared for ethical reasons, we allow authors to make their data sets available upon request. For information on unacceptable data access restrictions, please see <http://journals.plos.org/plosone/s/data-availability#loc-unacceptable-data-access-restrictions>.

Before we proceed with your manuscript, please address the following prompts:

a) If there are ethical or legal restrictions on sharing a de-identified data set, please explain them in detail (e.g., data contain potentially identifying or sensitive patient information, data are owned by a third-party organization, etc.) and who has imposed them (e.g., a Research Ethics Committee or Institutional Review Board, etc.). Please also provide contact information for a data access committee, ethics committee, or other institutional body to which data requests may be sent.

**RESPONSE: Unfortunately, de-identified data are not available for public access, according to the British Columbia Ministry of Health and the UBC Research Ethics Board. We have added this information in the disclaimer, page 2, line 29. A link to access data has been added on line 33.**

b) If there are no restrictions, please upload the minimal anonymized data set necessary to replicate your study findings to a stable, public repository and provide us with the relevant URLs, DOIs, or accession numbers. Please see <http://www.bmj.com/content/340/bmj.c181.long> for guidelines on how to de-identify and prepare clinical data for publication. For a list of recommended repositories, please see <https://journals.plos.org/plosone/s/recommended-repositories>. You also have the option of uploading the data as Supporting Information files, but we would recommend depositing data directly to a data repository if possible.

Please update your Data Availability statement in the submission form accordingly.

3. Your ethics statement should only appear in the Methods section of your manuscript. If your ethics statement is written in any section besides the Methods, please delete it from any other section.

**RESPONSE: We have now moved the ethical approval subsection to the end of the methods section, page 15, line 201.**

4. Please include captions for your Supporting Information files at the end of your manuscript, and update any in-text citations to match accordingly. Please see our Supporting Information guidelines for more information: <http://journals.plos.org/plosone/s/supporting-information>.

**RESPONSE: Done, as requested.**

5. If the reviewer comments include a recommendation to cite specific previously published works, please review and evaluate these publications to determine whether they are relevant and should be cited. There is no requirement to cite these works unless the editor has indicated otherwise.

**RESPONSE: Done, as requested.**

6. Please review your reference list to ensure that it is complete and correct. If you have cited papers that have been retracted, please include the rationale for doing so in the manuscript text, or remove these references and replace them with relevant current references. Any changes to the reference list should be mentioned in the rebuttal letter that accompanies your revised manuscript. If you need to cite a retracted article, indicate the article’s retracted status in the References list and also include a citation and full reference for the retraction notice.

**RESPONSE: Done, as requested.**

[Note: HTML markup is below. Please do not edit.]

Reviewers' comments:

Reviewer's Responses to Questions:

**Comments to the Author**

1. Is the manuscript technically sound, and do the data support the conclusions?

The manuscript must describe a technically sound piece of scientific research with data that supports the conclusions. Experiments must have been conducted rigorously, with appropriate controls, replication, and sample sizes. The conclusions must be drawn appropriately based on the data presented.

Reviewer #1: Partly

Reviewer #2: Yes

2. Has the statistical analysis been performed appropriately and rigorously?

Reviewer #1: No

Reviewer #2: N/A

3. Have the authors made all data underlying the findings in their manuscript fully available?

The [PLOS Data policy](http://track.editorialmanager.com/CL0/http:%2F%2Fwww.plosone.org%2Fstatic%2Fpolicies.action%23sharing/1/010f019c73ee6756-5d7e074b-5251-45bb-adb3-3cfc016b1e05-000000/6Da4OJ1Ds8AR6cQ22P6pC_tXGHEM0oPt8GY5JCWFQIo=251) requires authors to make all data underlying the findings described in their manuscript fully available without restriction, with rare exception (please refer to the Data Availability Statement in the manuscript PDF file). The data should be provided as part of the manuscript or its supporting information, or deposited to a public repository. For example, in addition to summary statistics, the data points behind means, medians and variance measures should be available. If there are restrictions on publicly sharing data—e.g. participant privacy or use of data from a third party—those must be specified.

Reviewer #1: Yes

Reviewer #2: Yes

4. Is the manuscript presented in an intelligible fashion and written in standard English?

PLOS ONE does not copyedit accepted manuscripts, so the language in submitted articles must be clear, correct, and unambiguous. Any typographical or grammatical errors should be corrected at revision, so please note any specific errors here.

Reviewer #1: Yes

Reviewer #2: Yes

5. Review Comments to the Author

Please use the space provided to explain your answers to the questions above. You may also include additional comments for the author, including concerns about dual publication, research ethics, or publication ethics. (Please upload your review as an attachment if it exceeds 20,000 characters)

**Comments from reviewer #1:**

**Comments for Manuscript PONE-D-25-53902**

I would like to commend the authors for their timely and important work in establishing a population-based cohort of mothers who use substances and their children in British Columbia. The study addresses a significant public health issue, and the cohort will serve as a valuable resource for future research aimed at improving maternal and child health outcomes in this vulnerable population. I also appreciate the editor for the opportunity to review this manuscript.

**General Comments**

The authors should carefully follow the **PLOS ONE manuscript preparation guidelines**. The current version lacks several required structural elements:

- The **Abstract** is not fully structured. It should clearly include *Methods, Results, and Conclusion* sections in addition to the Background/Objective.
- The main text does not contain a **separate, structured Discussion and Conclusion section**. At present, the manuscript ends abruptly without a clear synthesis of findings, interpretation and limitations
- The Discussion should be organized to:
  - Summarize the principal findings.
  - Compare results with existing literature.
  - Address strengths and limitations.
  - Provide implications for practice, policy, and future research.
- The Conclusion should be concise, highlighting the key message of the study.

Without these sections, the manuscript does not meet the journal’s formatting and reporting standards. A thorough restructuring is required to ensure compliance and improve readability.

**Response: We have now relabelled and otherwise reorganized the paper to conform to journal standards.**

Introduction

The introduction effectively sets the context by highlighting the rising rates of perinatal substance use (PSU) and associated adverse outcomes, particularly in British Columbia. The emphasis on systemic gaps in care and the impact of stigma is well-placed.

Suggestion: Consider including a brief mention of the intersectional vulnerabilities faced by Indigenous and racialized communities in BC, which are noted later in the manuscript but could be introduced earlier to frame the urgency of the research.

Question: The authors mention that PSU is under-researched due to ethical concerns and healthcare system failures. Could they elaborate on what specific ethical concerns have historically impeded research in this area?

**Response: disclosure and the potential for child removal.**

- **Refer to structural barriers surrounding PSU population impeding their participation in research, particularly mothers.**
- **Elaborate on impacts for other subgroups within this population (i.e., Indigenous people)**

Funding Statement Placement

The authors currently places the funding information at the end of the Introduction, which is not consistent with the PLOS ONE guidelines. According to the journal’s requirements, funding details should be presented under a separate heading (e.g., *Funding*) in the appropriate section of the manuscript, not embedded within the Introduction.

For example, instead of including text such as: *“Funding for this cohort was obtained from the Health Canada Substance Use and Addiction Program (2223-HQ-000028; 1819-HQ-000036).”*

at the end of the Introduction, the authors should move this statement to a dedicated Funding section (usually after the Acknowledgments or before Competing Interests).

Recommendation:

- Provide a clear heading (*Funding*) in line with PLOS formatting.
- Place the funding statement in the correct section, ensuring consistency with other required declarations (Competing Interests, Ethics Statement, Data Availability).
- Keep the Introduction focused on background and rationale, without administrative details

This applies to the Ethical Approval section as well.

**Response: Done, as requested.**

Methods

Cohort Identification and Substance Use Definition:

- The authors have used a 12-month window prior to the first pregnancy-related healthcare contact to identify substance use. While this approach aims to capture ongoing use, it may also include individuals with historical or sporadic use that is not necessarily active during pregnancy.
- Suggestion: Consider discussing the potential for misclassification and how this may affect the cohort’s representativeness. Sensitivity analyses using narrower time windows (e.g., during pregnancy only) could strengthen the validity of substance use identification.
- Question: Were any validation studies conducted to assess the accuracy of the case-finding algorithm in identifying active substance use during pregnancy?

**Response: Unfortunately, validation is not possible as no ‘gold standard’ measurement (ie. via self report) is feasible given the de-identified nature of the data. We have added the following to the discussion section (page 22, line 295): “*“Moreover, our classifications of substance use disorders and administrative loss to follow-up may result in misclassification. We will consider alternative thresholds to assess the robustness of results to these thresholds.”***

Data Linkage and Missing Information:

- The cohort links data from ten administrative databases, which is a strength. However, the absence of linkage to the BC Ministry of Child and Family Development is noted as a limitation, as it precludes identification of state-initiated child apprehensions.
- Suggestion: The authors should elaborate on how they plan to address this in future updates, and **whether partnerships with child welfare agencies are being** **pursued**.
- Question: What proportion of children in the cohort are estimated to be affected by child welfare involvement, and how might this bias the findings related to child separations?

**Response: We have pursued such partnerships and determined these data are not suitable for research purposes. Somewhat unbelievably, they are not collected systematically and availability of provincial health numbers are inconsistent. To our knowledge such data has never been used for research purposes.**

2. Loss to Follow-up and Censoring:
   - The authors define administrative loss to follow-up as no health system contact for ≥66 months. While empirically derived, this cutoff may not fully capture individuals who have moved out of province or who disengage from healthcare due to stigma or systemic barriers.
   - Suggestion: Consider discussing alternative methods to ascertain vital status or out-of-province migration, such as linkage to national databases or mortality registries.
   - Question: Were there differences in demographic or clinical characteristics between those lost to follow-up and those retained? If so, how might this affect longitudinal analyses?

**Response: Linkage to datasets held outside the province of BC is not currently possible; such actions could only be taken by the provincial government, as only de-identified data is provided to researchers. We have noted this as a limitation on page 22, line 295: *“Moreover, our classifications of substance use disorders and administrative loss to follow-up may result in misclassification. We will consider alternative thresholds to assess the robustness of results to these thresholds.”***

Measurement of Maternal-Child Separation:

- The use of geographic mismatches and social assistance records to infer separations is innovative but indirect. The authors note that they cannot distinguish between voluntary separations, kinship care, and foster care.
- Suggestion: Acknowledging this limitation is important, and future qualitative or mixed-methods studies could help contextualize these separations.
- Question: How were inconsistencies between separation indicators (e.g., SDPR vs. geographic mismatch) resolved, and what is the estimated agreement between these measures?

**Response: This work is currently underway – we did not elaborate on details as we are assessing not only concordance between separation indicators, but also assessing use of geographic indicators from different component datasets within our linked data. We intend to report on this effort more exhaustively in forthcoming manuscripts, we have added the following (page 13, line 188*): “Later separations can be inferred based on the geographic location of the mother and child in their individual health records (available in multiple component datasets; subject to evaluations of concordance) or by assessing whether a mother-child pair are attached to the same social assistance record.”***

**Overall**
The methods are generally robust and well-described, and the cohort represents a significant contribution to perinatal substance use research. Addressing the above points will enhance the clarity, validity, and utility of the cohort profile for future users.

Results

- The descriptive tables are comprehensive and well-organized. The stratification by substance type is particularly useful.
- Notable Findings: The high prevalence of mental health disorders among mothers (8%) and children (26.3%), along with the substantial rates of maternal mortality (3.6%) and child separation (60%), are striking and underscore the cohort's public health relevance.
- Suggestion: In Table 1, consider presenting the proportion of mothers with polysubstance use (e.g., opioids + stimulants) to better characterize the complexity of substance use patterns.
- Question: The median follow-up time varies substantially by substance type (e.g., 6 years for cannabis-only vs. 13 years for "other substances"). What might explain these differences, and how will the analyses account for varying follow-up durations?

**Response: RE: the suggestion – given the large extent of overlap and potential for many additional columns we chose to maintain the current table structure (which was in fact reduced from prior iterations in which combinations were considered!). Re: the question, we believe the difference in cannabis reporting in particular is attributable to legalization in Canada in 2018, which would have increased reporting.**

**Comments from reviewers #2:**

Reviewer #2: Review of "Cohort Profile: Mothers who use substances and their children in British Columbia, Canada"

16 February 2026

Lines 71-84
The authors might consider adding a brief description of substance treatment that exists outside the medical model, e.g. 12 Step and other peer-to-peer recovery programs, for clarity, and whether this is part of the studies being planned. Notably, this type of peer-to-peer delivery of services is a significant support for many people trying to recover from substance use disorders.

**Response:** We have now referenced our environmental scan of existing services in BC on page 5, line 88.

Line 133
Consider changing "Tobacco use was not evaluated." to "Tobacco and caffeine use was not evaluated." or something similar, for additional clarity, and to help destigmatize drug use by highlighting that the majority of Canadian adults use substances, e.g. caffeine.

**Response: Done, as requested. We have used the term nicotine to also capture vaping and other forms of use.**

Table 2
The table has two asterisked footnotes. Consider switching one of these to a different symbol, for clarity.

**Response: Done, as requested.**

Lines 278-279
Consider including indigenous and immigrant status in the list of social factors that may affect health outcomes, for clarity, and to highlight these differentials.

**Response: while an indicator of First Nations status is available in our data, this is the only indicator of race/ethnicity, and no fields for immigration status are available. We have also been instructed not to use this field by our colleagues at the First Nations Health Authority as it may further stigmatize people from First Nations communities.**

Lines 301-304
The authors might consider adding a description of difficulty in including peer-to-peer recovery support below the text related to harm reduction services, for clarity.

**Response: Done, as requested on page 23, line 317: *“Further linkage to community and local services is necessary to characterize health care service utilization, including the use of peer support services.”***

Supplemental Table 2
In the footnote, consider changing "... , or In the care of ..." to "... , or in the care of ..." to correct a typographical error.

**Response: Done, as suggested.**
